# Factors Associated with Coverage and Usage of Long-Lasting Insecticidal Nets in Madagascar

**DOI:** 10.1155/2009/451719

**Published:** 2010-01-26

**Authors:** Neeta Thawani, Manisha A. Kulkarni, Salim Sohani

**Affiliations:** ^1^Canadian Red Cross, 170 Metcalfe Street, Ottawa, ON, Canada K2P 2P2; ^2^HealthBridge, 1 Nicholas Street, Ottawa, ON, Canada KIN 7B7

## Abstract

In October 2007, long-lasting insecticidal nets (LLINs) were distributed in 59 of the 111 districts in Madagascar as part of a nationwide child survival campaign. A community-based cross-sectional survey was conducted six months post-campaign to evaluate net ownership, use and equity. Here, we examined the effects of socioeconomic factors on LLIN ownership and usage in districts with and without net distribution during the campaign. Our data demonstrated that in districts with LLIN distribution, LLIN ownership was similar across all wealth groups in households with at least one child under the age of five years (90.5% versus 88.6%); in districts without net distribution, 57.8% of households in the poorest tertile compared to 90.1% of households in the least poor tertile owned at least one LLIN. In contrast, in LLIN-owning households, both in districts with and without net distribution, higher socio-economic status was not associated with use among children under five years. These findings suggest that socio-economic status contributes to the household net ownership but once a household owns a net, socio-economic status is not associated with net use.

## 1. Introduction

Malaria continues to be a major cause of morbidity and mortality among young children and pregnant women living in endemic areas. Approximately 40% of the world's population, primarily those living in the world's poorest countries, is at risk of malaria, resulting in about 250 million clinical cases and more than one million deaths annually [1]. Current treatment and vector control interventions to combat malaria include artemisinin-based combination therapy (ACT), intermittent preventive treatment in pregnancy (IPT), indoor residual spraying of insecticide (IRS), and insecticide-treated nets (ITNs). However, ACT and IPT use are still very low. According to national household surveys carried out in African countries in 2006-2007, use of ACT by young children was found to be approximately 3% on average while use of IPT by pregnant women was 18% on average, reinforcing the need to enhance prevention efforts against malaria [1]. ITNs are a key prevention tool that have been found to reduce malaria cases by 50% and decrease all-cause mortality in young children by 15%–30% in controlled efficacy trials, where coverage rates are high [2, 3]. However, when implemented as part of national malaria control programs, the challenge lies in selecting delivery method(s) that result in high coverage among vulnerable populations from all socio-economic backgrounds. Maintaining high coverage and usage limit parasite transmission and are essential for achieving community-wide protection [4, 5].

In many countries, insecticide-treated nets and/or long-lasting insecticidal nets (LLINs) have been distributed through a combination of delivery systems including routine health services, commercial sources, subsidized social marketing, and free mass campaigns [6–9]. In particular, free mass distribution campaigns, which have been the chosen method for distribution of vaccines, de-worming medications and other necessary supplements, have achieved high coverage and typically reach over 90% of children uniformly across all socioeconomic backgrounds [10]. With free integrated ITN/LLIN distribution and mass vaccination campaigns, many countries have seen dramatic increases in treated net coverage [9, 11]. Although many still believe that high rates of ITN coverage can be brought about by social marketing and routine health services [12], Noor et al. have found that free mass distribution achieves the highest ITN coverage among the poorest households, ensuring equitable distribution of ITNs [13]. Understanding the socio-economic factors that affect coverage and usage rates will provide insight into selecting appropriate delivery methods to ensure equitable access to nets.

Half of Madagascar's population is at high risk of malaria infection [1]. To reduce morbidity and mortality in children under the age of five years, particularly due to measles and malaria, the Ministry of Health, with the Madagascan Red Cross and many world-wide partners, organized a nationwide integrated child survival campaign to provide immunization against measles and distribution of vitamin A tablets, mebendazole and LLINs [14]. Over one and a half million LLINs were distributed to children under five years in 59 (south and west) of the 111 districts at the household level at mobile distribution points in each fokotany (smallest administrative unit). The distribution strategy was one LLIN per child under five years and up to a maximum of two LLINs per household. LLINs were also provided to pregnant women at distribution points in roughly half of the 59 districts. Since the eastern zone of Madagascar had previously benefited from a total of 2 million nets through routine healthcare services such as pre-natal care clinics for pregnant women and immunization visits for children under the age of five years as well as subsidized prices through social marketing distributions in 2006, this region was not targeted to receive LLINs during the campaign.

In Madagascar, where close to 70% of its population lives below the poverty line, this division provided a unique opportunity to investigate the impact of socio-economic factors on coverage and usage in areas with LLIN integration, where the primary source of nets was the integrated campaign, and areas without LLIN integration, where the primary source of nets was commercial sources, such as shops and kiosks. A nationwide cross-sectional survey was conducted six months post-campaign. Here, we examined the effects of wealth, urban/rural status and mother's level of education on LLIN ownership and usage in areas with and without LLIN integration during the child survival campaign.

## 2. Methods

### 2.1. Study Design

To assess household ownership and usage of nets, a survey was conducted six months after the integrated campaign, from 11 April to 1 May 2008, during the high season of malaria transmission. A community-based, two-stage cluster sampling method was used followed by simple random sampling of households. The main outcome used for the sample-size calculations is the proportion of children under the age of five years sleeping under an ITN the previous night 6 months after the distribution campaign. The survey was designed to have at least 90% power to estimate ITN usage by children under five years within a range of 5%, assuming a non-response rate of 15%, a design effect of 2 and that 66% of the households have a child under the age of five years.

The country's 111 districts were divided into three areas: 26 districts where the Madagascan Red Cross distributed LLINs during the campaign, 33 districts where the Ministry of Health distributed LLINs during the campaign and 52 districts where LLIN distribution was not integrated into the campaign. In each area, 10 districts were selected using the probability proportional to size (PPS) methodology. Using PPS, six *fokontany* (smallest administrative unit) were selected from each district. Since some *fokontany* were inaccessible at the time of the survey due to flooding caused by hurricanes, alternate fokontany were selected on the basis of PPS in the respective districts. Sampling was based on projected 2008 population estimates extrapolated from the most recent census which took place in 2003 (INSTAT, 2003). All households in a *fokontany* were listed using a personal digital assistant (PDA) equipped with an internal global positioning system (GPS) and GPS Sample software, previously described by Vanden Eng et al., was used to randomly select 24 households and 6 replacement households to be surveyed [15]. A total of 4,320 households were included in this survey.

The surveyors conducted interviews with any capable adult household member to collect information on the number of high-risk individuals (children under five years and pregnant women) and their net ownership and usage practices as well as all women of reproductive age. The surveyors asked questions about the household: pregnant women, children under five years, ownership and usage of nets, and the economic characteristics of the household. Net ownership and net brand were confirmed by observation as well as by information on the campaign card received during the campaign week.

### 2.2. Definitions

Areas with LLIN integration include the 59 districts in the west and south where LLINs were distributed during the integrated campaign. Areas without LLIN integration include the 32 east coast districts where LLINs were not distributed during the integrated campaign. The 20 central highland districts, where malaria transmission is irregular and LLINs are not included in the national strategy against malaria, were excluded from estimates for districts without LLIN integration during the campaign. Households are defined as “all persons who eat out of the same food pot and recognized the same head of household”. Coverage is defined as the proportion of households possessing at least one LLIN. Usage is defined as the proportion of children that were reported to have slept under an LLIN the previous night. Children under five years were defined as children aged 0–59 months at the time of the campaign. The wealth of each household was determined using economic indicators and asset scores from the 2004 Madagascar Demographic and Health Survey [16]. All households from both areas were ranked on the basis of the wealth score and subsequently divided into tertiles. This ensured a common wealth classification of households in both areas while providing sufficient sample size for statistical comparison of wealth groups within each area. Households ranked within the lowest third of scores were designated the poorest tertile; those within the second third were designated as poor and those within the highest third as the least poor tertile. Individual *fokontany* were classified as urban or rural by officials at the Madagascar Ministry of Health, Family Planning and Social Protection. Mothers indicated their education level as no education, completion of primary, secondary level I, secondary level II, above secondary or do not know. For the purpose of this analysis, mother's education level was ranked as no education, completion of primary or secondary and above.

### 2.3. Statistical Analysis

Data analyses were performed using SAS version 9.1 (SAS Institute, Cary, NC, USA). The PROC SURVEYFREQ function was used to estimate proportions and 95% confidence intervals taking into account clustering at the fokontany level and stratified by operational zone. We used multivariate logistic regression with the PROC SURVEYLOGISTIC function to assess the association between coverage and usage and the following factors: wealth status, urban/rural status and mother's level of education. Estimates and standard errors were weighted based on the probability of being selected. A Rao-Scott chi square was used to test for differences in proportions. Statistical significance was defined as *P* < .05.

### 2.4. Ethical Clearance

The survey was conducted with the understanding and informed consent of all respondents and caregivers. The survey was approved by Madagascar Ministry of Health, Family Planning and Social Protection, Antananarivo, Madagascar and the institutional review boards of HealthBridge, Canada and the Centers for Disease Control and Prevention, Atlanta, GA.

## 3. Results

### 3.1. LLIN Coverage of Households with Children under Five Years and Socioeconomic Status

4302 households were surveyed (refusal rate 1.5%). 1698 households in areas with LLIN integration during the campaign and 418 households in areas with no LLIN integration had at least one child under five years. LLIN coverage among households with at least one child under the age of five years at the time of the campaign was 90.0% (CI 87.6–92.5) in areas with LLIN integration and 77.4% (CI 71.7–83.2) in areas with no LLIN integration. Table 1 shows the distribution of LLIN ownership by households with children less than five years during the campaign by wealth status, urban/rural status and mother's level of education. In areas with LLIN integration, coverage was similar among households in the poorest and least poor wealth tertiles (90.5% versus 88.6%). Urban/rural status or mother's level of education was not associated with LLIN ownership in these areas.

In areas with no integration, LLIN coverage was substantially lower among households with at least one child under the age of five years in the poorest tertile compared to the least poor tertile (57.8% versus 90.1%; adjusted odds ratio, AOR = 0.1 CI 0.1–0.3; *P* < .0001). There was no association between coverage and urban/rural status (AOR = 1.1 CI 0.6–2.0) or mother's level of education (AOR, primary level = 1.2 CI 0.5–2.9; AOR, secondary level and above = 1.0 CI 0.5–2.3).

### 3.2. LLIN Usage by Children under Five Years and Socioeconomic Status

In areas with LLIN integration, 2369 children under five years were surveyed; in areas with no LLIN integration, 523 children under five years were surveyed. In households with at least one LLIN, the proportion of children under five years of age who slept under an LLIN the previous night was high in both areas with and without integration; 94.6% (CI 92.9–96.2) in districts with LLIN integration and 90.0% (86.2–93.7). As shown in Table 2, usage of LLINs by children was slightly higher in the poorest tertile compared to the least poor tertile (96.8 versus 90.9%; AOR = 3.2 CI 1.8–5.7; *P* = .0001) in areas with integration. There was no association between usage of LLINs by children and urban/rural status (AOR = 1.3 CI 0.5–3.6) or mother's level of education (AOR, primary level = 0.8 CI 0.3–1.6; AOR, secondary level and above = 0.8 CI 0.4–1.9) in areas with integration.

In the absence of net integration, we found no association between usage of LLINs by children and wealth status (AOR, poorest tertile = 1.4 CI 0.4–4.4), urban/rural status (AOR = 0.8 CI 0.2–2.8) or mother's level of education (AOR, primary level = 0.7 CI 0.3–2.0; AOR, secondary level and above = 1.7 CI 0.4–6.3).

## 4. Discussion

Here, we demonstrated that free mass distribution of LLINs allowed for ownership of LLINs equally among people at risk regardless of their socio-economic status. Similar levels of LLIN ownership among households with children under five years was evident across economic tertiles in districts with LLIN integration during the campaign (poorest tertile: 90.5%, least poor tertile: 88.6%), in areas without LLIN integration, LLIN ownership was higher in the least poor households with children under five years (poorest tertile: 57.8%, least poor tertile: 90.1%). However, in households that owned at least one LLIN, both in areas with and without net integration during the campaign, higher socio-economic status was not significantly associated with use among children under five years. These findings suggest that free mass distribution allows equitable ownership of nets; and once a household acquires a net(s), they are highly likely to use them regardless of their socio-economic status.

Madagascar is among the world's poorest countries with 68.7% of its population living below the poverty income level of approximately 45 cents a day according to its National Institute of Statistics (INSTAT, 2005). Families can barely afford enough food to meet their basic caloric needs, and expenses such as buying a net as well as associated transaction costs are a choice to buy less food and increase hunger. Our data demonstrated that in areas with no LLIN integration, where the major sources of LLINs were commercial, LLIN ownership was the highest in the least poor households. Indeed, in a study in Uganda by Nuwaha, where nets were available primarily through commercial sectors, higher wealth was found to be associated with ownership of bed nets [17]. Moreover, in a survey conducted in Nigeria, lower socio-economic groups were found to be the least likely to pay for nets [18]. While it is important to keep in mind that delivery of ITNs through subsidized commercial sources has shown some success in improving ITN coverage [19], eliminating costs by distributing ITNs through free mass campaigns is an effective method to achieve high coverage rates with a high level of equity. Similar to wealth status, we found that LLIN ownership was similar in urban and rural areas in areas with integration. Moreover, in areas without integration, LLIN ownership was higher in urban than rural areas. However, after adjusting for wealth status and mother's level of education, urban/rural status was not associated with LLIN ownership, suggesting that asset-based wealth status was the major factor influencing LLIN ownership.

Contrary to the effects of socio-economic status on LLIN ownership, LLIN usage was not associated with higher wealth status both in areas with and without LLIN integration during the campaign. Rather, in areas with LLIN integration, usage by children under five years was slightly higher in poorer households that owned an LLIN, suggesting that although the poorest households received LLINs free of charge, it is valued as an effective malaria prevention strategy. In fact, in a study designed to assess if nets provided free of charge in villages in Tanzania will be cared for by owners, Maxwell et al. found that 90% of the nets were retained and brought back to be re-treated years later [20]. Possible reasons such as a greater nuisance of insects, the lack of additional malaria prevention tools such as window netting, and targeting of the poorest through social mobilization efforts before and after LLIN distribution campaigns may explain why occupants of poorer households are more likely to use bed nets [21]. Other than wealth status, we found no association between LLIN usage and urban/rural status or mother's level of education in both areas with and without integration. Earlier published reports have shown a disparity between socio-economic factors, such as education level, and usage of nets. Evidence from social marketing in Malawi revealed that only 3.3% of rural children under five years compared to 24% of urban children slept under a net the previous night [22]. Moreover, Noor et al. demonstrated that homestead wealth, travel time to nearest market and mother's education were associated with the use of bed nets by children under five years of age [13]. However, recent reports, which are consistent with our results, have suggested that net usage is not necessarily determined by higher socio-economic status. Goesch et al. demonstrated that in Gabon, the percentage of net users was significantly higher among families in the lowest group of the economic score [21]. Furthermore, in a recent analysis of fifteen standardized national surveys from 2003 to 2006, Eisele et al. found that socioeconomic status was not a significant factor associated with ITN use among children in households that own an ITN [23]. Taken together, these results indicate that higher socio-economic status may influence ITN use through access to ITNs but is not directly associated with ITN use among children in households that own an ITN.

This analysis had several limitations. First, there was no baseline study conducted pre-campaign to determine LLIN ownership and usage in areas with and without net integration. As a result, valid comparisons of post-campaign LLIN ownership and usage between areas with and without LLIN integration were not possible and the analysis was therefore restricted to within-area comparisons. Second, the number of households was insufficient to permit division of households into wealth quintiles, as is commonly done [9, 18]. Hence, households were divided into tertiles on the basis of wealth scores. It is possible that fine differences in wealth status are masked by this treatment; however, households that would otherwise fall into the intermediate wealth quintiles are included in our analysis in both the poorest and least poor tertiles, which would be expected to diminish any differences between groups. The observed differences in our analyses thus may underestimate the actual differences between wealth groups.

In conclusion, our findings provide evidence that introducing a cost barrier to ITN distribution does not allow for equitable access to ITNs. ITNs have been proven to be an effective prevention tool for malaria by protecting both the person(s) sleeping under the net as well as the community as a whole. However, in order to achieve community-wide protection by preventing parasite transmission by mosquitoes, ITNs must be accessible and used widely within a community, even by those who cannot afford to purchase them.

## Figures and Tables

**Table 1:** Factors associated with ownership of at least one LLIN in households with children aged 0–59 months at the time of the campaign in districts with LLIN integration and districts without LLIN integration during the campaign^1, 2^.

	Ownership (%; 95% CI)	Odds ratio (95% CI)	Adjusted odds ratio (95% CI)
*Areas with integration*			
Wealth status			
Poorest (*n* = 710)	90.5 (87.1–93.9)	1.2 (0.7–2.1)	1.3 (0.6–2.7)
Poor (*n* = 569)	91.7 (88.5–94.9)	1.6 (0.9–2.8)	1.6 (0.9–3.1)
Least poor (*n* = 419)	88.6 (83.9–93.2)	Reference	Reference
Urban/rural status			
Rural (*n* = 1612)	90.7 (88.1–93.3)	1.5 (0.8–2.8)	1.5 (0.7–3.1)
Urban (*n* = 86)	85.0 (78.2–91.8)	Reference	Reference
Education level of mother			
Primary (*n* = 842)	90.6 (87.7–93.4)	0.9 (0.6–1.4)	1.0 (0.6–1.5)
Secondary and above (*n* = 367)	89.1 (84.7–93.5)	0.8 (0.4–1.5)	0.9 (0.5–1.8)
None (*n* = 580)	91.3 (87.6–95.0)	Reference	Reference
*Areas with no integration*			
Wealth status			
Poorest (*n* = 113)	57.8 (45.2–70.4)	0.2 (0.1–0.3)	**0.1 (0.1**–**0.3)**
Poor (*n* = 122)	76.4 (68.8–84.0)	0.4 (0.2–0.7)	**0.4 (0.2**–**0.7)**
Least poor (*n* = 183)	90.1 (86.6–93.6)	Reference	Reference
Urban/rural status			
Rural (*n* = 346)	74.1 (67.5–80.8)	0.4 (0.2–0.8)	1.1 (0.6–2.0)
Urban (*n* = 72)	89.7 (86.6–93.0)	Reference	Reference
Education level of mother			
Primary (*n* = 256)	78.6 (69.9–87.3)	1.7 (0.8–3.8)	1.2 (0.5–2.9)
Secondary and above (*n* = 115)	86.2 (81.3–91.1)	2.9 (1.5–5.6)	1.0 (0.5–2.3)
None (*n* = 103)	68.0 (55.9–80.2)	Reference	Reference

^1^Abbreviations: CI = confidence interval

^2^Significant adjusted odds ratios are indicated in bold

**Table 2:** Factors associated with usage of LLINs by children aged 0–59 months given that the household had at least one LLIN in districts with LLIN integration and districts without LLIN integration during the campaign^1, 2^.

	Use (%; 95% CI)	Odds ratio (95% CI)	Adjusted odds ratio (95% CI)
*Areas with integration*			
Wealth status			
Poorest (*n* = 860)	96.8 (95.3–8.4)	3.1 (1.8–5.4)	**3.2 (1.8**–**5.7)**
Poor (*n* = 723)	95.5 (93.5–97.6)	2.2 (1.2–3.9)	**1.9 (1.0**–**3.6)**
Least poor (*n* = 489)	90.9 (87.6–94.1)	Reference	Reference
Urban/rural status			
Rural (*n* = 1959)	94.8 (93.1–96.5)	1.7 (0.6–4.6)	1.3 (0.5–3.6)
Urban (*n* = 93)	91.5 (84.0–98.9)	Reference	Reference
Education level of mother			
Primary (*n* = 982)	94.4 (92.1–96.6)	0.6 (0.3–1.3)	0.8 (0.3–1.6)
Secondary and above (*n* = 377)	93.8 (90.5–97.1)	0.5 (0.2–1.2)	0.8 (0.4–1.9)
None (*n* = 671)	96.6 (94.2–99.0)	Reference	Reference
*Areas with no integration*			
Wealth status			
Poorest (*n* = 85)	91.1 (81.6–100.0)	1.2 (0.3–4.3)	1.4 (0.4–4.4)
Poor (*n* = 105)	88.0 (81.4–94.5)	0.8 (0.3–2.0)	1.1 (0.4–2.7)
Least poor (*n* = 176)	90.0 (83.8–95.9)	Reference	Reference
Urban/rural status			
Rural (*n* = 292)	88.6 (83.8–93.4)	0.6 (0.1–2.8)	0.8 (0.2–2.8)
Urban (*n* = 69)	92.4 (82.3–100.0)	Reference	Reference
Education level of mother			
Primary (*n* = 218)	86.4 (81.5–91.3)	0.7 (0.3–1.9)	0.7 (0.3–2.0)
Secondary and above (*n* = 99)	92.7 (84.8–100.0)	1.6 (0.3–8.2)	1.7 (0.4–6.3)
None (*n* = 84)	89.9 (80.4–99.4)	Reference	Reference

^1^Abbreviations: CI = confidence interval

^2^Significant adjusted odds ratios are indicated in bold
